# First Line of Defense: The Vital Role of Family Medicine Physicians in Preventing Acute Mesenteric Ischemia in High-Risk Patients

**DOI:** 10.7759/cureus.40359

**Published:** 2023-06-13

**Authors:** Sara Arfan, Lavanyah Anbazhagan, Frederick Tiesenga

**Affiliations:** 1 General Surgery, Windsor University School of Medicine, Chicago, USA; 2 General Surgery, West Suburban Medical Center, Chicago, USA

**Keywords:** cost burden, cardiovascular prevention, general surgery, family medicine, non-occlusive mesenteric ischemia

## Abstract

Acute mesenteric ischemia (AMI) is a life-threatening condition with a high mortality rate despite surgical interventions. Morbidity and mortality are especially high in those with risk factors, which include diabetes mellitus (DM), hypertension (HTN), coronary artery disease, recent myocardial infarction, and rheumatic autoimmune diseases, among others. We present the case of a 70-year-old Caucasian woman diagnosed with AMI. The patient presented acutely to the emergency department after nine episodes of vomiting and was admitted to the surgical floor the same day for an emergent exploratory laparotomy. She presented acutely with an atypical presentation and without any progressive symptoms, despite various comorbidities. This patient was classified as "very high risk", but she had not been on any medications or monitored for any of her comorbidities. We highlight the essential and multifaceted role of family medicine physicians, also known as primary care physicians (PCPs), in the prevention of bowel ischemia and recommend the use of routine outpatient monitoring with clinical examination, blood testing, and imaging. These, along with a high index of suspicion, have clinical utility in preventing hospitalization, surgical intervention (bowel resection), and other serious sequelae of AMI. Timely detection, management, and specialist referrals from a family medicine physician can lower the overall burden on healthcare resources and personnel.

## Introduction

Acute mesenteric ischemia (AMI) also known as abdominal apoplexy, is commonly referred to as a "bowel attack" [1]. It is rather uncommon; however, women are three times more likely than men to get AMI [2]. Acute mesenteric ischemia results from an inadequate blood supply to the affected bowel segment [3]. Despite a low incidence (rare in outpatient clinical settings and comprising only 0.09% to 0.2% of emergency department admissions) [4], it is a life-threatening condition with a mortality rate of 60% to 80% even with surgical interventions [5]. Morbidity and mortality are especially high in those with risk factors and must be recognized and treated emergently [6]. Risk factors include diabetes mellitus (DM), hypertension (HTN), coronary artery disease, recent myocardial infarction, rheumatic autoimmune diseases, and iatrogenic causes (post-bypass surgery from dislodgement of intravascular devices) [7,8]. 

Management of AMI requires a multidisciplinary team of physicians, starting with family medicine physicians, who have a crucial role in the prevention and management of patients with AMI. Family medicine physicians are the first point of contact for many patients. And in the case of patients with AMI, routine monitoring and follow-ups can identify those at high risk and manage their underlying comorbidities. They can also provide evidence-based lifestyle recommendations and timely referrals to other specialists, such as cardiologists and gastroenterologists. Recent literature findings suggest that AMI has specific diagnostic radiologic features characteristic of different pathologic stages [9]. Therefore, routine imaging can be used as an indicator of disease severity and can help with earlier detection at a reversible disease stage, preventing irreversible bowel necrosis and resection. A high degree of clinical suspicion through focused history combined with routine imaging in the outpatient setting can prove useful for prevention and timely management [10].

## Case presentation

A 70-year-old Caucasian woman presented to the emergency department at a community hospital complaining of abdominal discomfort for six hours. The patient had severe acute periumbilical abdominal pain and intermittent cramping, which eventually spread diffusely through the entire abdomen. The pain presented acutely during a bowel movement and was later accompanied by nausea, malaise, and nine episodes of non-bilious emesis. There was no post-prandial abdominal pain, aversion to meals, melena, hematochezia, diarrhea, or constipation. A review of systems was otherwise negative. Past medical history was significant for coronary artery disease (CAD), DM, HTN, hyperlipidemia (HLD), rheumatoid arthritis, and Sjogren syndrome. The patient has had previous hospitalizations for placement of coronary artery bypass graft (CABG) and angiography with stent placement for 98% right coronary artery (RCA) occlusion. She also experienced a silent myocardial infarction (MI) and had a history of premature ventricular contractions (PVCs). The patient also reported a history of "some nerve damage in the colon" and stated that she normally has a bowel movement every three to four days. She was not on any medications nor was she being monitored by a family medicine physician. Family history was significant for heavy tobacco use, stroke, multiple atrial fibrillation episodes in the mother, and death from rheumatoid heart disease in the father. 

On physical examination, there was no rebound tenderness, guarding, or rigidity. Admission vitals were within normal limits. Laboratory testing of complete blood count (CBC) revealed elevated levels of leukocyte count (13.3 k/mm cu), erythrocyte sedimentation rate (ESR) at 58 mm/hr, serum glucose of 216 mg/dL, prothrombin time of 19.6 sec, international normalized ratio (INR) of 1.7, partial pressure of carbon dioxide (pCO2) of 51.9 mmHg, and troponin 0.04 ng/mL. Serum levels of calcium, albumin, and lipase were decreased at 8.4 mg/dL, 3.1 g/dL, and 3 IU/L, respectively (Table 1).

**Table 1:** Patient’s laboratory results on admission CBC: Complete blood count, ESR: Erythrocyte sedimentation rate, pCO2: Partial pressure of carbon dioxide, INR: International normalized ratio, CA-125: Cancer antigen-125, ANA: Antinuclear antibody

Laboratory investigation	Patient's results	Reference range	Interpretation
CBC			
Leukocyte count	13.3	4.0-11.0 k/mm cu	Elevated
ESR	58	0-30 mm/hr	Elevated
Serum glucose	216	70-99 mg/dL	Elevated
pCO2	51.9	35-45 mmHg	Elevated
Lipase	3	11-82 IU/L	Decreased
Hypercoagulable work-up			
Prothrombin time	19.6	10.3-12.4 sec	Elevated
INR	1.7	0.9-1.2	Elevated
Protein C	112	73%-180%	Normal
Protein S	60	63%-140%	Decreased
Factor V Leiden	Heterozygous (one factor V Leiden or PT 20210 gene copy)	Heterozygous (normal) Homozygous (abnormal)	Normal
CA-125	94.9	0-35 U/mL	Elevated
ANA	Positive		Abnormal
Beta-hydroxybutyrate	7.3	0.2-2.8 mg/dL	Elevated
Vitamin B12	1800	232-1245 pg/mL	Elevated

**Figure 1:**
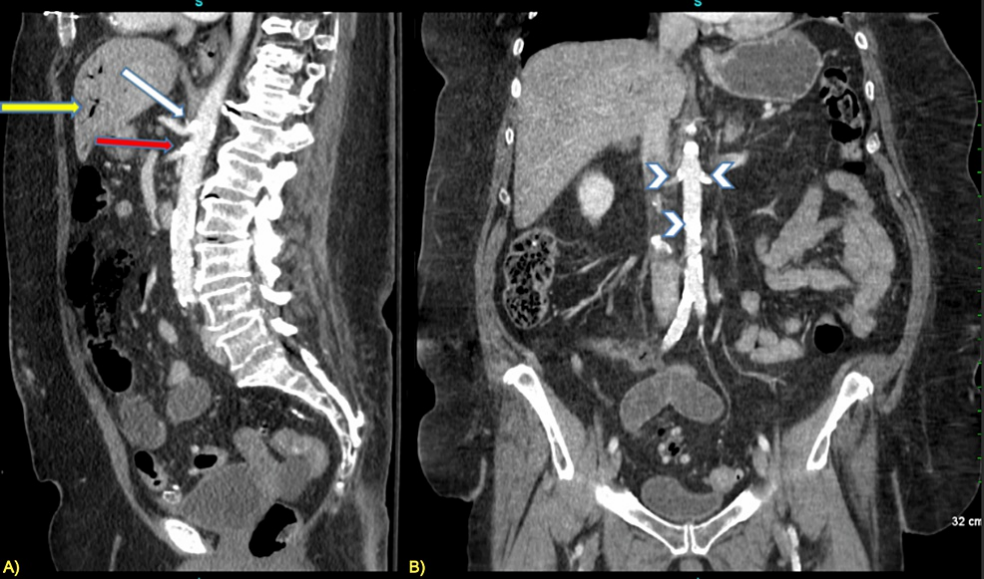
Sagittal view of the abdomen A) Portal venous gas (yellow arrowhead), densely calcified plaques at the ostium of the celiac artery (white arrowhead), and small mesenteric artery (red arrowhead) B) Several calcified plaques in the abdominal aorta (white arrowheads)

**Figure 2:**
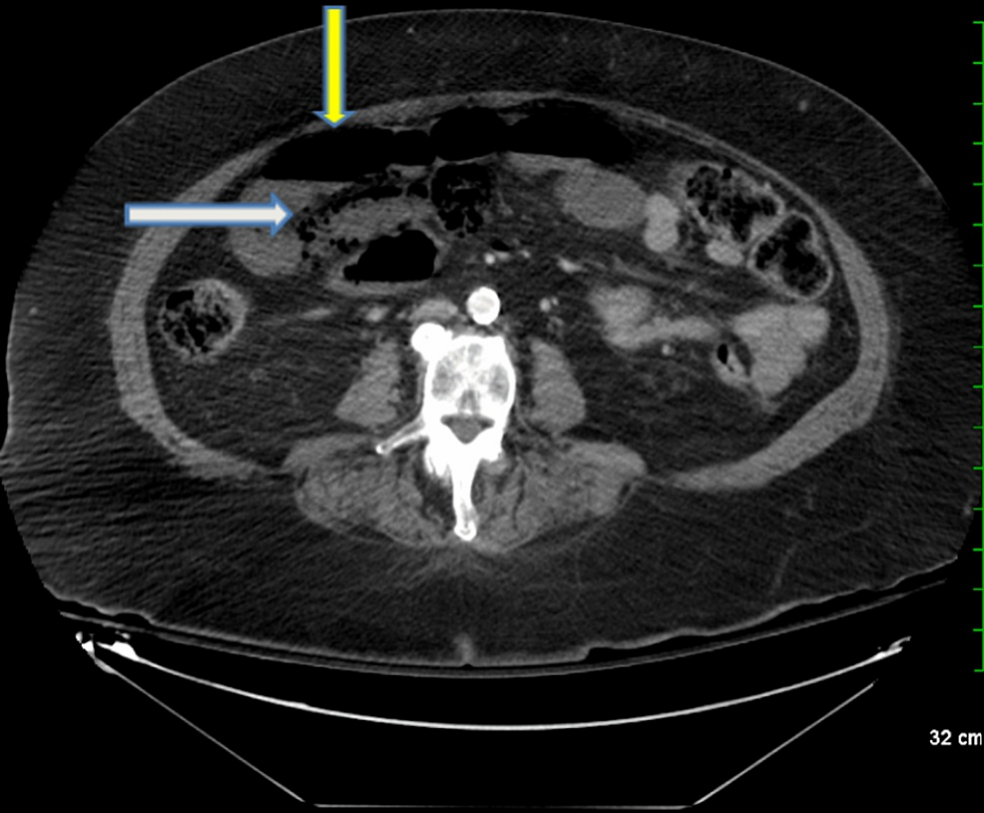
Axial view of CT demonstrating pneumatosis (white arrowhead) in a bowel loop (white arrowhead) and dilated loops of bowel (yellow arrow)

A CT with IV contrast (Figure 1) revealed a calcified celiac artery and superior mesentery artery (SMA). Several calcified plaques in the abdominal aorta were also appreciated (Figure 1). Additional CT findings revealed pneumatosis in a bowel loop among other dilated loops of bowel (Figure 2). 

**Figure 3:**
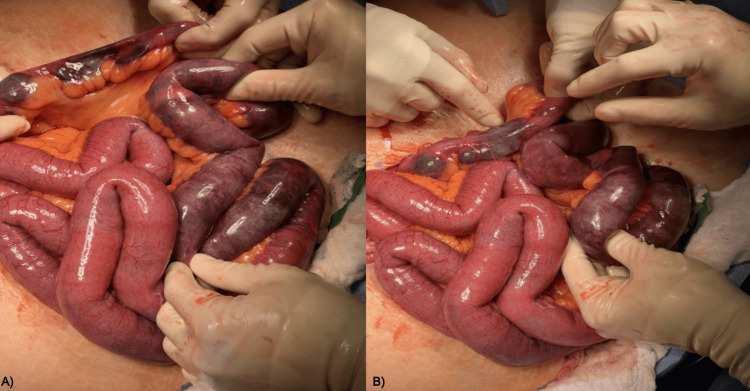
Intraoperative images A) Gross specimen of the ischemic terminal ileum, demonstrated intraoperatively; B) Opacified ischemic terminal ileum with enteric contents upstream

**Figure 4:**
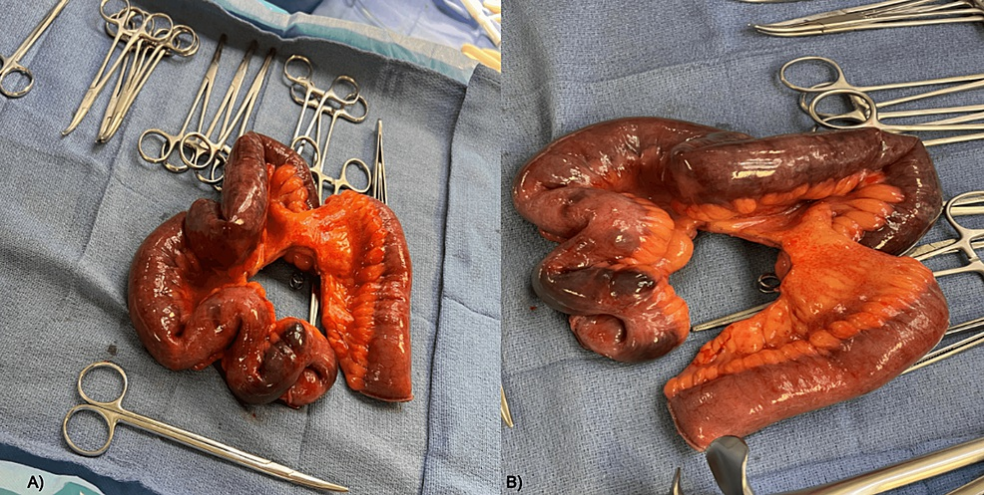
Images of the resected segment A) Gross resected segment of ischemic terminal ileum; B) Observed are streaks of dark mucous and segmental thinning without perforation, fibrosis, strictures, or superficial vascular lesions

**Figure 5:**
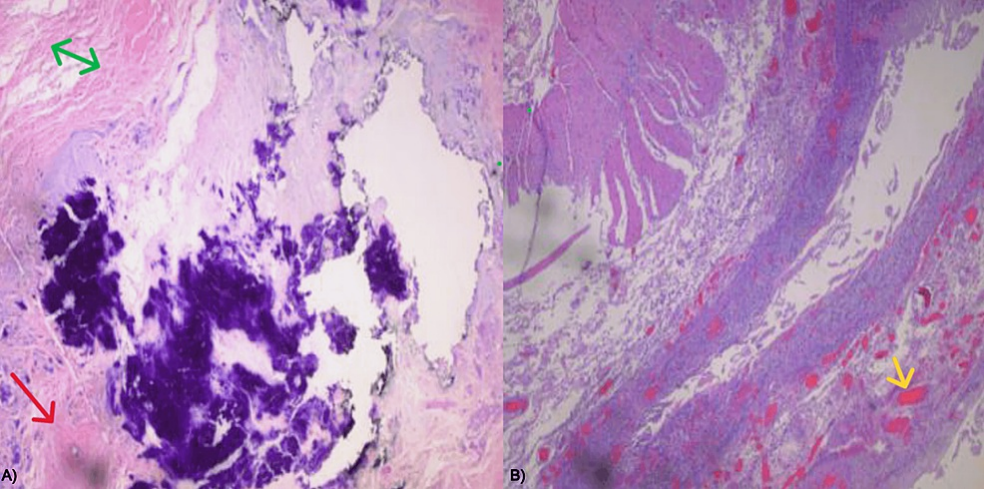
Pathology specimen of umbilical stalk cyst A) Fibro-adipose tissue with dense fibrosis (green arrowhead), dystrophic calcification, epidermal inclusion cyst (red arrowhead), and reactive changes; B) Ischemic and hemorrhagic changes (yellow arrowhead) with focal acute and chronic inflammation along with reactive changes

A diagnosis of ischemic bowel was made and the patient was transferred from the emergency department to the surgical floor for an emergency exploratory laparotomy. Intraoperatively, the ischemic terminal ileum was located (Figure 3) and resected (Figure 4). An umbilical stalk cyst and an abdominal mass were excised and sent for pathology (Figure 5), which demonstrated fibro-adipose tissue with dystrophic calcification, epidermal inclusion cyst, and reactive changes. The pathologic specimen was negative for malignancy. 

Postoperatively, the patient was provided antibiotics, prophylactic low-dose molecular weight heparin (LMWH), and dual antiplatelet therapy with clopidogrel and aspirin. She was discharged on postoperative day five, with a Jackson-pratt (JP) drain in place, and was counseled on the importance of closely following up with her PCP.

## Discussion

Acute mesenteric ischemia is due to an inadequate blood supply to a segment of the bowel, resulting in life-threatening complications [3]. It can result from arterial embolism, arterial thrombosis, non-occlusive mesenteric ischemia, venous thrombosis, mesenteric vasculitis, or mesenteric artery dissection [11]. Due to a wide range of etiologies, it has overlying presentations with other gastrointestinal pathologies, resulting in missed or delayed diagnosis. Physician awareness in combination with timely utilization of available diagnostic and interventional tools is key to detecting AMI and decreasing the associated morbidity and mortality.

The largest percentage of AMI cases (50%) is due to an arterial embolism; whereby the presentation results from a cardiac source, namely myocardial infarction, endocarditis, or ventricular arrhythmias [12]. This results in the formation of a mural thrombus which can embolize the mesenteric arteries. The SMA, which exits at an acute, oblique angle from the aorta, is where the majority of visceral arterial emboli tend to lodge [13]. In this scenario, symptom onset is often dramatic due to poor formation of collateral vasculature. Typically, patients present with an abrupt onset of severe abdominal pain with hematochezia [14].

In contrast to the abrupt process, acute mesenteric arterial thrombosis accounts for less frequent ischemic events (typically 25% to 30 %)[12]. The frequent cause is an in-situ atherosclerotic disease; most commonly near the origin of the SMA. Besides atherosclerotic disease processes, inflammatory rheumatological disease states predispose to vasculitic changes in the blood vessels and are an independent cause of SMA thrombosis. The gradual nature of this disease process results in the development of collateral circulation over time. This results in bowel ischemia once the last collateral artery is occluded. As a result, the size of the intestinal infarct and the degree of the bowel infarction is generally greater compared to the accompanying embolic phenomenon [11]. Superior mesenteric artery thrombosis has greater than 70% mortality compared to SMA embolization due to delayed diagnostic detection, resulting in an extensively ischemic bowel which requires complex revascularization procedures [11]. Thus, the management of AMI requires a multidisciplinary approach.

Our patient presented with acute periumbilical abdominal pain and intermittent cramping. The diffuse pain presented acutely during a bowel movement and was later accompanied by nausea, malaise, and non-bilious emesis. Symptoms indicative of chronic intestinal insufficiency such as postprandial discomfort, nausea, and weight loss are common prodromal symptoms seen in patients with SMA. In addition to the traditional risk factors of atherosclerosis (HTN, HLD, DM), this patient also had elevated markers of inflammation (elevated ESR) which are associated with her underlying autoimmune disorders, Rheumatoid arthritis and Sjogren's disease, for which she was not being treated. By adopting a strong clinical suspicion and an assertive approach in patients presenting with risk factors for bowel ischemia (advanced age, cardiovascular diseases, diabetes, prior abdominal surgery, inflammatory rheumatological conditions), early identification and control of risk factors can be preventative of a disease which continues to have high mortality.

Recent evidence suggests the clinical utility and prognostic value of biomarkers like D-dimer and L-lactate for patients with AMI [15]. Other lines of evidence suggest using radiographic evidence to stage the disease [9,10]. Primary care physicians can benefit from these latest advances in research and order routing blood tests, imaging, and other diagnostic tests to closely monitor the disease stage and progression to prevent and/or treat accordingly. Prevention of AMI should be based on a comprehensive personalized treatment/prevention plan which should take into account the patient's medical history and risk factors. Lastly, PCPs can also make timely referrals to other specialists that could potentially avoid hospitalization or surgical intervention down the line. Inpatient admission for AMI not only places a burden on the healthcare resources and personnel but also on the patient from complications of surgical intervention, which include infection, adhesions, and nutritional deficiencies [12]. A successful outcome for patients at risk of AMI is timely detection, prevention, and management, which can be easily achieved in an outpatient setting. We recommend the use of routine clinical examination, blood testing, and imaging along with clinician awareness and suspicion to have clinical utility in preventing a bowel resection or other serious sequelae of AMI.

## Conclusions

Primary care physicians are the first point of contact for many patients as their initial symptoms surface. It is crucial for PCPs to identify high-risk patients and closely monitor their underlying comorbidities with routine visits to decrease the risk of ischemia. This can be done by managing medications and recommending the implementation of lifestyle modification strategies to reduce overall risk. Primary care physicians must have a high index of suspicion for patients with risk factors for AMI, which include smoking, physical inactivity, and a high saturated-fat diet. The risks are highest in patients from lower socioeconomic backgrounds with poor access to healthcare resources, resulting in poor preventative care that requires patient education on smoking cessation, physical inactivity, and dietary changes at routine outpatient visits. A review of medications is also crucial, as the implementation of prophylactic antiplatelet and anticoagulant medications is known to prevent AMI. However, the use of these medications must be carefully balanced with the patient's risk of bleeding, which requires tactful monitoring by a PCP in the outpatient setting.
